# Differences in the expression of chromosome 1 genes between lung telocytes and other cells: mesenchymal stem cells, fibroblasts, alveolar type II cells, airway epithelial cells and lymphocytes

**DOI:** 10.1111/jcmm.12302

**Published:** 2014-05-15

**Authors:** Xiaoru Sun, Minghuan Zheng, Miaomiao Zhang, Mengjia Qian, Yonghua Zheng, Meiyi Li, Dragos Cretoiu, Chengshui Chen, Luonan Chen, Laurentiu M Popescu, Xiangdong Wang

**Affiliations:** a Department of Pulmonary Medicine, Fudan University, Zhongshan Hospital, Shanghai Respiratory Research InstituteShanghai, China; b Department of Pulmonary Medicine, The First Affiliated Hospital, Wenzhou Medical UniversityWenzhou, China; c Biomedical Research Center, Fudan University Zhongshan Hospital and Qinpu HospitalShanghai, China; d Fudan University Center for Clinical BioinformaticsShanghai, China; e State Key Laboratory of Systems Biology, Chinese Academy of ScienceShanghai, China; f Division of Cellular and Molecular Medicine, Carol Davila University of Medicine and PharmacyBucharest, Romania; g Department of Molecular Medicine, Victor Babeş National Institute of PathologyBucharest, Romania; h Division of Advanced Studies, Victor Babeş National Institute of PathologyBucharest, Romania

**Keywords:** chromosome 1, genes, lung, telocytes, mesenchymal stem cells, fibroblasts, alveolar type II cells, airway epithelial cells, lymphocytes

## Abstract

Telocytes (TCs) are a unique type of interstitial cells with specific, extremely long prolongations named telopodes (Tps). Our previous study showed that TCs are distinct from fibroblasts (Fbs) and mesenchymal stem cells (MSCs) as concerns gene expression and proteomics. The present study explores patterns of mouse TC-specific gene profiles on chromosome 1. We investigated the network of main genes and the potential functional correlations. We compared gene expression profiles of mouse pulmonary TCs, MSCs, Fbs, alveolar type II cells (ATII), airway basal cells (ABCs), proximal airway cells (PACs), CD8^+^ T cells from bronchial lymph nodes (T-BL) and CD8^+^ T cells from lungs (T-LL). The functional and feature networks were identified and compared by bioinformatics tools. Our data showed that on TC chromosome 1, there are about 25% up-regulated and 70% down-regulated genes (more than onefold) as compared with the other cells respectively. Capn2, Fhl2 and Qsox1 were over-expressed in TCs compared to the other cells, indicating that biological functions of TCs are mainly associated with morphogenesis and local tissue homoeostasis. TCs seem to have important roles in the prevention of tissue inflammation and fibrogenesis development in lung inflammatory diseases and as modulators of immune cell response. In conclusion, TCs are distinct from the other cell types.

## Introduction

Telocytes (TCs) have been identified in multiple tissues/organs, including heart, liver, kidneys, uterus, skin, intestine, trachea, lungs and others [1–20], as a unique type of cell, different from Fbs and fibroblast-like cells [21]. There is still a challenge to determine and develop TC-specific biomarker(s) useful for TCs identification in tissues/organs, as the features of TCs are obvious only by transmission electron microscopy (TEM). For instance, podoms and podomers can be discriminated only by TEM.

Telocytes form a 3D network to contact with different kinds of cells within tissues/organs (*e.g*. blood vessels, nerve bundles, resident cells or immunoreactive cells) [1,8,22–25]. For example, TCs were found to be present near the basement membrane of the bronchiolar epithelium, among or surrounding airway smooth muscle cells, or in the pulmonary interstitial space [23,24,26]. The intercellular communication and long-distance signalling are assured by homocellular junctions between TCs and heterocellular junctions between TCs and neighbour cells, *e.g*. tissue-specific cells, immunocompetent cells and putative stem cells [22,25]. In addition, the immunophenotypical and electrophysiological features and the specific microRNA signatures of TCs were also explored [10,14,21,27,28].

Previously, we have identified and isolated TCs from the trachea and lung tissues of the mouse [24,29], and compared the variation of gene expression profiles among pulmonary TCs, stem cells and Fbs to demonstrate the distinction of TCs from other cells with, similar location [30,31]. Furthermore, the proteomic profile of human lung TCs revealed differences between these cells and Fbs and emphasized that TCs play an important role in extracellular environment homoeostasis, tissue morphogenesis, development and repair/remodelling [31,32].

This study aims to start the investigation of features and patterns of TC-specific gene profiles and signatures in chromosomes, look at the network of most important genes and explore the potential functional associations. We compared gene expression profiles of pulmonary TCs, MSCs, Fbs, ATII cells, ABCs and PACs. We also included lymphocytes from bronchial lymph nodes (T-BL) and from lungs (T-LL), because they might interact with TCs in the lung and trachea. The functional and characteristic networks were identified and compared by bioinformatics tools.

## Material and methods

### Data collection and mining

We obtained gene expression profiles of pulmonary TCs on days 5 and 10, MSCs, Fbs from previous study [30]. ATII, ABCs, PACs, T-BL and T-LL gene expression profiles were obtained from the National Center for Biotechnology Information (NCBI) Gene Expression Omnibus database (GSE6846, GSE27379, GSE28651) [33]. The microarray was composed of 45,101 probes. Our first filter eliminated the probe sets without corresponding official symbol, leaving 39,417 probes and 21,680 genes.

### Cell lines and tissue sampling

Telocytes were isolated from BALB/c mouse lung tissue and cultured for 5 and 10 days, respectively. Mesenchymal stem cells and fibroblast cell lines were obtained from Sciencell Research Laboratories (Cat. no. M7500-57, Carlsbad, CA, USA) and from Chinese Academy of Science (Cat. no.GNM28, Shanghai, China), respectively. Alveolar type II cells (GSM157835-GSM157837) were isolated from 8 weeks old sex age matched littermate control mice. CD8+ T-cells from lungs and bronchial lymph nodes (GSM677065) and CD8+ T-cells from bronchial lymph nodes, lymphocytes (GSM677065) derived from mice (CL4). Murine proximal airway duct (GSM709834-GSM709836) and basal cells (GSM709832, GSM709833) were isolated from 8-12 week old C57BL/6 mice.

Our earlier study investigated the gene expression profile, composed of 23,861 probes, of mouse lung TCs, Fbs and MSCs [30]. After eliminating the probes without corresponding official symbol, there are 13,236 probes and 11,532 genes. Only those genes whose expressions have been measured in all the studies were considered in our analysis. In total, 11,532 genes were analysed and 645 genes of the chromosome 1 were focused and furthermore analysed in the present study.

### Data processing

Let matrix *B* represents the expression of whole genes, the row represents a gene, and the column represents a cell. Matrix *H* represents the expression of housekeeping gene. The average expression of each cell was calculated, and a new vector *Y* was defined according to the formulation 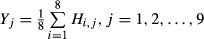. The average value of the first four cell mean gene expression was calculated as variable *C*, and 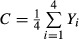. Another new vector *Z* was calculated, where *Z*_*i*_ = 1, *i* = 1, 2, …, 4 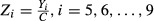, and matrix *E*, where 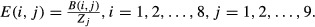


### Identification of differentially expressed genes

Expression data were normalized through quantile normalization and the Robust Multichip Average (RMA) algorithm included in the NimbleScan software. The Probe level (*_norm_RMA.pair) files and Gene level (*_RMA.calls) files were generated after normalization. All gene level files were imported into Agilent GeneSpring GX software (version 11.5.1) for further analysis. Differentially expressed genes between two samples were identified through Fold Change filtering. Hierarchical clustering was performed using the Agilent GeneSpring GX software (version 11.5.1). Gene Ontology (GO) analysis and Pathway analysis were performed using the standard enrichment computation method. The propensity of functional changes was reflected in different levels of the gene expression in diverse cell types. The mouse has 20,000–25,000 genes, about 85% similar to the human homolog. The present study used the gene expression profiles of different mouse cells to seek TC-specific genes and explore the function of TCs. Fold changes were utilized to identify differentially expressed genes or simply differential genes. Up- or down-regulated folds of TC genes were calculated on basis of the comparison with other cells, after the averages of gene expression in cells were obtained from the raw data of multi-databases, as shown in Table S1. The fold changes were calculated according to the formula as following: (the density of gene expression in TCs—the density of gene expression in another cell)/ the density of gene expression in TCs.

## Results

Gene expression array data showed that 14 genes (Ralb, Igsf8, Sdpr, Csrp1, Uck2, Rab3gap2, Arpc2, Nav1, Psmd1, Tagln2, Tpp2, Capn2, Fhl2, Qsox1) in chromosome 1 of TCs were up-regulated (> 1 fold), as compared with the other cells (Table 1). Among them, calpain 2 (Capn2), four and a half LIM domains 2 (Fhl2) and quiescin Q6 sulfhydryl oxidase 1 (Qsox1) were over-expressed two- to fivefold in both TC5 and TC10, as compared with the other cells (Table 1B). Thirty-nine genes in TCs were down-regulated, as compared with the other cells (Table 2) and, among them, two genes, transcription elongation factor A (SII) 1 (Tcea1) and interferon activated gene 203 (Ifi203) were expressed twofold lower in TCs than in the other cells. The majority of genes down-regulated in TCs contribute to the nucleoplasm and membrane-enclosed compartments.

**Table 1:** Summary of genes expressed preferentially in TCs, as compared with others

Compaired pairs/fold up-regulated	>1	>2	>5
TC5 *versus* others	36	5	0
TC10 *versus* others	32	9	0
TCs *versus* others	14	3	0

	Folds (TC5 *versus* others/TC10 *versus* others)						
	
Gene symbol	Fibroblast	Stem	ATII	CD8_T_BL	CD8_T_LL	Basal_cell	Duct_cell
(A) Genes up-regulated between one- and twofold in TCs as compared with others							
Arpc2	3.83/3.24	3.3/2.8	2.46/2.85	1.64/1.96	1.55/1.82	3.05/3.65	2.33/2.78
Csrp1	28.43/17.04	13.87/8.31	3.24/2.66	228.4/192.8	170.88/142.28	1.96/1.66	1.77/1.49
Igsf8	4.34/4.58	2.54/2.68	1.38/1.99	5.63/8.36	3.18/4.65	1.92/2.86	2.54/3.76
Nav1	2.18/1.81	1.22/1.01	1.76/2	3.68/4.31	1.39/1.6	1.4/1.64	2.12/2.47
Psmd1	2.9/1.74	2.73/1.64	1.58/1.3	2.44/2.07	3.16/2.64	2.36/2	2.08/1.76
Rab3gap2	1.85/2	1.98/2.15	2.55/3.78	1.52/2.32	1.21/1.82	6.15/9.41	4.3/6.55
Ralb	2.26/1.15	5.23/2.65	2.48/1.72	20.44/14.6	12.82/9.03	1.76/1.26	1.89/1.35
Sdpr	1577.05/1478.54	2.43/2.28	1.91/2.45	135.46/178.89	70.18/91.42	1.31/1.73	3.58/4.72
Tagln2	25.31/13.65	8.37/4.52	1.37/1.01	3.97/3.01	3.49/2.61	6.86/5.23	5.33/4.03
Tpp2	1.45/1.03	3.33/2.37	5.62/5.48	2/2	1.68/1.66	2.29/2.3	2.15/2.15
Uck2	2.72/1.86	1.56/1.06	6.07/5.67	4.9/4.72	8.43/8.01	5.71/5.51	3.43/3.29

(B) Genes up-regulated between two- and- fivefold in TCs as compared with other							
Capn2	4.45/2.75	8.92/5.5	3.59/3.03	8.46/7.34	8.9/7.62	3.05/2.65	2.98/2.58
Fhl2	142.78/104.63	12.96/9.49	2.95/2.96	212.64/219.48	15.09/15.37	10.39/10.75	3.34/3.44
Qsox1	6.8/4.12	3.44/2.09	5.04/4.18	44.91/38.34	29.66/24.97	2.37/2.03	3.08/2.63

**Table 2:** Summary of genes expressed preferentially in TCs, as compared with others

Compaired pairs/fold down-regulated	>1	>2	>5
TC5 *versus* others	66	5	1
TC10 *versus* others	127	20	2
TCs *versus* others	39	2	1

	Folds (TC5 *versus* others/TC10 *versus* others)						
	
Gene symbol	Fibroblast	Stem	ATII	CD8_T_BL	CD8_T_LL	Basal_cell	Duct_cell
(A) Genes down-regulated between one- and twofold in TCs as compared with others							
1700025G04Rik	1.11/1.35	1.94/2.37	1.23/1.1	2.89/2.51	2.89/2.54	3.47/3	2.85/2.48
3110009E18Rik	2.51/2.52	1.14/1.14	7.44/5.46	9.53/6.79	5.58/4.03	18.98/13.5	7.34/5.25
4930558K02Rik	2.53/3.61	2.42/3.45	1.52/1.59	3.97/4.03	2.39/2.45	3.03/3.07	3.47/3.53
Ankzf1	2.46/3.23	1.22/1.6	1.99/1.9	6.46/6.01	4.42/4.17	3.52/3.26	2.42/2.26
Copa	1.12/1.64	1.25/1.83	3.68/3.95	3.03/3.17	1.74/1.84	1.73/1.8	1.82/1.9
Cps1	1.25/1.78	1.16/1.65	6.79/7.07	3.27/3.3	7.27/7.45	15.41/15.54	13.13/13.31
Dgkd	2.01/3.58	1.59/2.84	1.44/1.87	3.23/4.08	2.36/3.03	2.96/3.73	1.64/2.08
Dpp10	1.93/2.93	1.22/1.85	1.6/1.77	3.55/3.82	3.02/3.29	4.4/4.73	5.06/5.46
Fam123c	1.12/1.62	1.11/1.62	3.41/3.62	1.64/1.69	12.07/12.59	6.87/7.06	1.12/1.16
Farp2	1.25/1.24	3.68/3.67	6.18/4.5	2.89/2.05	2.22/1.59	8.38/5.91	7.61/5.4
Farsb	1.5/1.53	1.69/1.72	2.99/2.23	4/2.9	2.52/1.85	3.16/2.28	3.48/2.53
Fmo4	1.33/2.22	2.25/3.76	2.2/2.69	1.38/1.63	3.66/4.4	8.29/9.81	2.42/2.88
Hspd1	2.28/3.4	1.3/1.94	2.35/2.57	1.31/1.39	2.28/2.45	3.43/3.63	5.91/6.29
Ivns1abp	1.18/1.75	1.27/1.88	2.51/2.73	5.18/5.46	6.92/7.39	12.9/13.57	17.99/19.02
Khdrbs2	1.09/1.2	1.18/1.3	28.85/23.21	21.96/17.16	5.16/4.09	10.45/8.15	5.91/4.63
Mfsd4	4.91/9.58	5.23/10.21	1.91/2.72	1.03/1.43	1.03/1.44	5.64/7.79	2.63/3.66
Myog	1.09/1.32	1.04/1.26	3.24/2.89	10.45/9.03	2.49/2.19	23.4/20.18	27.04/23.44
Nek2	3.24/3.46	1.79/1.9	5.16/4.02	2.32/1.75	3.48/2.67	7.2/5.44	7.79/5.91
Nfasc	1.27/2.15	1.2/2.03	1.37/1.68	2.49/2.98	3.25/3.94	4.7/5.62	3.23/3.88
Nr5a2	1.38/1.09	1.54/1.22	6.37/3.68	4.87/2.73	11.26/6.4	23.97/13.42	8.45/4.76
Obsl1	3.06/3.32	1.17/1.27	4.79/3.79	8.26/6.36	3.54/2.76	22.9/17.59	12.76/9.85
Ogfrl1	1.2/1.53	1.58/2.02	6.21/5.81	2.55/2.32	2.32/2.14	19.25/17.46	13.46/12.27
Parp1	1.49/1.88	1.22/1.53	11.54/10.6	12.53/11.17	8.95/8.1	7.45/6.63	6.49/5.81
Pogk	3.47/3.72	1.23/1.32	3.28/2.57	6.86/5.22	6.78/5.23	5.73/4.35	4.87/3.72
Rbbp5	1.34/1.6	1.38/1.65	16.55/14.41	36.45/30.84	23.18/19.88	17.62/14.88	11.54/9.79
Rev1	1.19/1.24	2.03/2.11	13.57/10.33	42.67/31.56	54.99/41.23	31.56/23.29	23.49/17.42
Rnf2	1.19/2.1	1/1.77	1.03/1.33	3.19/3.99	2.29/2.9	1.52/1.9	1.76/2.21
Rps6kc1	1.19/1.49	1.17/1.47	24.09/22.19	6.93/6.2	1.16/1.05	19.1/17.05	28.26/25.36
Rqcd1	1.16/1.2	1.01/1.05	23.94/18.13	29.07/21.38	16.82/12.54	17.36/12.74	18.95/13.98
Sf3b1	2.06/2.09	1.64/1.66	131.21/97.24	459.15/330.54	486.78/355.27	185.16/133.01	197.36/142.51
Tbc1d8	1.33/1.51	1.23/1.4	180.09/149.99	4.21/3.41	5.74/4.71	330/266.4	310.95/252.32
Tmem198	2.26/3.11	2.31/3.19	2.09/2.1	1.95/1.9	2.66/2.63	13.49/13.16	5.02/4.92
Tram1	1.91/1.95	1.01/1.03	21.7/16.14	25.36/18.32	15.65/11.46	26.04/18.78	17.81/12.91
Trip12	1.07/1.44	1.28/1.73	1.64/1.62	4.53/4.35	4.29/4.17	2.92/2.79	2.53/2.43
Trpa1	1.04/1.64	1.17/1.85	6.47/7.49	13/14.6	5.24/5.96	24.72/27.72	9.03/10.17
Vamp4	1.43/1.09	1.52/1.16	5.36/2.99	15.13/8.21	14.26/7.85	7.12/3.85	5.35/2.91
Zfp451	1.22/1.32	1.71/1.85	3.5/2.77	10.13/7.78	9.22/7.18	5.57/4.27	4.25/3.27

(B) Genes down-regulated between two- and fivefold in TCs as compared with others							
Tcea1	3.47/3.92	2.32/2.62	5.73/4.72	6.43/5.15	4.8/3.9	13.48/10.77	12.42/9.97

(C) Genes down-regulated between 5- and 10-fold in TCs as compared with others							
Ifi203	56.9/33.79	112.55/66.85	13.97/6.06	163.54/68.95	126.56/54.1	24.98/10.51	78.94/33.39

A set of genes are specifically up- or down-regulated in pulmonary TCs, as compared with the other cells (Table 3). The number of up- and down-regulated genes more than onefold in TCs at 5th day was 491 and 154, 412 and 233, 144 and 501, 98 and 547, 120 and 525, 166 and 479, or 166 and 479, as compared with MSCs, Fbs, ATII, ABCs, PACs, T-BL or T-LL respectively. The number of up- and down-regulated genes more than onefold in TCs at 10th day was 390 and 255, 317 and 328, 148 and 497, 106 and 539, 124 and 521, 166 and 479, or 170 and 475, as compared with MSCs, Fbs, ATII, ABCs, PACs, T-BL or T-LL respectively. The number of up- and down-regulated genes more than onefold in TCs at 5th day as well as at 10th day was 377 and 110, 305 and 221, 124 and 477, 86 and 527, 82 and 505, 147 and 460, or 148 and 457, as compared with MSCs, Fbs, ATII, ABCs, PACs, T-BL or T-LL respectively. Details of up- and down variations of genes on chromosome 1, including the number and names of up- and down-regulated genes more than onefold among different cells, are listed in Table S2.

**Table 3:** The number of genes specifically up- or down-regulated in pulmonary telocytes, as compared with other cells respectively

Compaired pairs	up>1	up>2	up>5	down>1	down>2	down>5	down>10
TC5 *versus* stem	491	228	58	154	44	7	4
TC10 *versus* stem	390	151	46	255	83	16	9
TCs *versus* stem	377	131	35	110	32	7	3
TC5 *versus* fibroblast	412	180	65	233	62	11	5
TC10 *versus* fibroblast	317	126	50	328	108	19	6
TCs *versus* fibroblast	305	110	43	221	58	8	3
TC5 *versus* ATII	144	67	13	501	397	228	125
TC10 *versus* ATII	148	72	17	497	400	211	115
TCs *versus* ATII	124	50	9	477	369	187	99
TC5 *versus* CD8BL	166	102	60	479	386	233	143
TC10 *versus* CD8BL	166	106	63	479	379	219	127
TCs *versus* CD8BL	147	89	49	460	356	198	115
TC5 *versus* CD8LL	166	90	54	479	386	241	144
TC10 *versus* CD8LL	170	99	51	475	378	233	129
TCs *versus* CD8LL	148	83	39	457	355	209	116
TC5 *versus* basal cell	98	43	13	547	463	331	219
TC10 *versus* basal cell	106	54	17	539	468	318	216
TCs *versus* basal cell	86	30	10	527	442	301	194
TC5 *versus* duct cell	120	57	13	525	440	289	178
TC10 *versus* duct cell	124	66	18	521	439	281	171
TCs *versus* duct cell	82	28	5	505	413	255	153

**Fig. 1:**
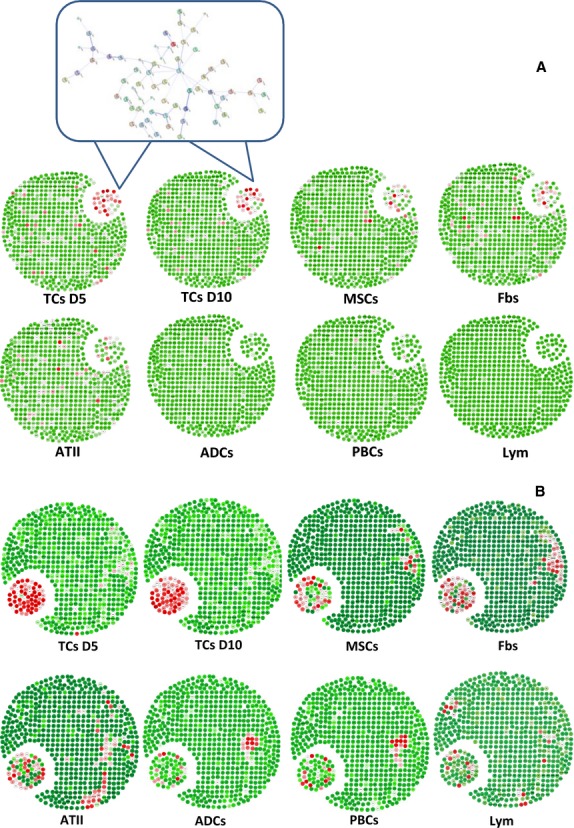
Expression profiles of the selected genes as an active group on chromosome 1 of telocytes (TCs), isolated and cultured from mouse lungs, on days 5 (D5) and 10 (D10), as compared with mesenchymal stem cells (MSCs), fibroblasts (Fbs), alveolar type II (ATII), airway basal cells (ABCs), proximal airway cells (PACs), lymphocytes (Lym) from bronchial lymph nodes and lung respectively (**A**). The profiles for entire genes are described in Data S1. The selected core network and whole mouse network are linked by the documented functional interactions from various databases (see Material and methods). Genes in each network are indicated in red and some of their nearest neighbours are indicated by grey nodes. A group of TC genes up- or down-regulated more than onefold as compared with the other cells and present in TCs on days 5 and/or 10 was selected as TC-specific genes. Top 61 up- or down-regulated genes of each cell were also evaluated and their distribution within chromosome 1 genes showed the difference between cells (**B**). Details of the selected network in each cell type are in Figures S1–9.

The relationships, including direct (physical) and indirect (functional) associations, of selected genes of TC chromosome 1 were analysed by String Network analysis (http://www.string-db.org). TC-specific genes were selected as a group of genes present in TCs on days 5 and/or 10, which were up- or down-regulated more than onefold as compared with all other cells. Figure 1A demonstrates the distribution of such active gene group on chromosome 1 of all selected cells, and the interaction and potential functional links of those genes in TCs. About 10–20% of those TCs genes showed the similar patterns of the expression in MSCs, Fbs or ATII, while few similarities were found between TCs and ABCs, PACs, T-BL or T-LL respectively. Top 100 up- or down-regulated genes of each cell line were also evaluated and their distribution within chromosome 1 genes showed significant difference between the cell lines, as shown in Figure 1B. Highly expressed genes on chromosome 1 of each cell line are evaluated and distributed in red colour (Fig. 1B). The distribution of the highly expressed genes of TCs at 5th and 10th day indicates that they are centred on the small cluster and different from the other cells. Among the 14 co-expressed genes (Table 1A and B), four genes were found to have certain interactions, as shown in Figure S1.

**Fig. 2:**
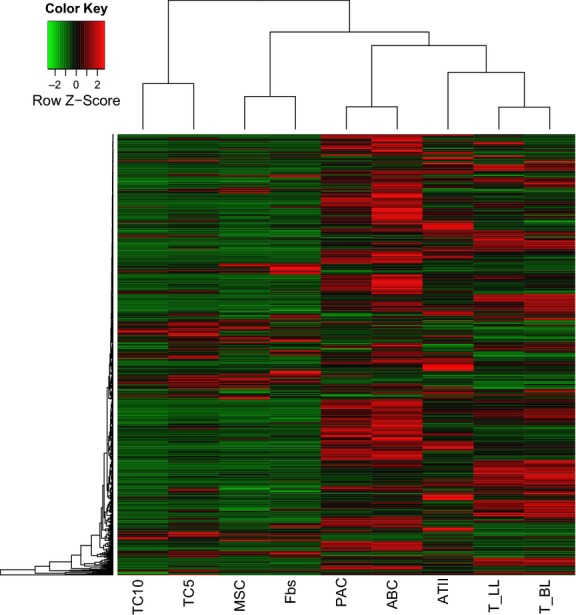
Hierarchical cluster analysis of the differentially expressed genes among telocytes (TCs), mesenchymal stem cells (MSCs), fibroblasts (Fbs), proximal airway cells (PACs), airway basal cells (ABCs) alveolar type II cells (ATII), and lymphocytes from lungs (T-LL) and bronchial lymph nodes (T-BL).

The hierarchical cluster analysis of the differentially expressed genes (Fig. 2) clearly shows that TCs are least related to the other cell lines.

## Discussion

Chromosome 1 is the largest autosomal chromosome, both in human and in mouse species, containing 3141 genes and 991 pseudo genes, representing about 8% of the total DNA in human cells [34]. Over 350 known diseases were proposed to be associated with genes on chromosome 1 [34].

In the present study, we analysed, for the first time, by bioinformatics tools, 650 genes of chromosome 1 from mouse in different cell types. We compared gene expression profiles of pulmonary TCs, MSCs, Fbs, ATII, ABCs, PACs, T-BL and T-LL. The main result is that 14 up-regulated or 39 down-regulated genes were found on chromosome 1 in TCs.

Three genes, Capn2, Fhl2 and Qsox1, were over-expressed in TCs compared with the other cells. The Capn2 gene encodes the calpain-2 protein, a member of non-lysosomal calcium-activated neutral proteases [35], involved in cell migration in response to calcium signalling [36]. Disruption of the Capn2 gene could result in embryonic death prior to implantation, demonstrating that Capn2 plays an important role in embryogenesis [37]. Such finding of Capn2 expression in TCs supports our previous hypotheses that TCs are involved in morphogenesis and tissue homoeostasis [30,31] and may have stronger protective effects against lung inflammation and injury as suggested by other results [38–40].

Fhl2 gene encodes four and one half LIM domain protein 2, reported to be a regulator in numerous signalling pathways. FHL2 protein interacts with plasma membrane integrins and plays the role of transcriptional coactivator after nuclear translocation [41]. We speculate that it may be one of key proteins from TCs able to interact with numerous lung resident cells, regulating signalling cascades and gene transcription through the sphingosine-1-phoshate signalling and activation of RhoA [42]. Therefore, TCs enriched with Capn2 and Fhl2 may be associated with the regulation of tissue inflammation, injury, repair, immune responses or cell movement [43–46].

Telocytes might be one of the key players in preventing the development of inflammation and fibrogenesis in chronic lung inflammation, since bleomycin-induced lung fibrosis is suppressed by FHL2 by attenuating lung inflammation [47]. Also, there are studies which incriminate TCs network loss in remarkable changes in tissue architecture [12,48] and fibrosis [49].

Qsox1 gene encodes Quiescin sulfhydryl oxidase 1, a protein involved in the oxidative protein folding, cell cycle control and extracellular matrix remodelling [50]. Studies on murine tissues showed that QSOX1 is over- expressed by foetal ecto- and mesodermal-derived tissues being related with extracellular matrix modelling, while in adults it enhances protein secretion [51]. In line with these findings, TCs could have stronger capacity of cell expansion, movement or proliferation. High amounts of QSOX1 were reported in breast, pancreas and prostate cancers [52] therefore we can consider TCs as important players in the maintenance of oxidative microenvironment preventing tumourigenesis [53].

The lowest expression of chromosome 1 genes in TCs, as compared with other cells, was found for Ifi203 (interferon activated gene 203) and Tcea1 (transcription elongation factor A SII-1). Ifi203 is the member of IFN-inducible gene family involved in innate immune responses, chronic inflammation and autoimmunity [54]. The low expression of Ifi203 in TCs suggested that they are involved in the immune response possibly by offering informative support to inflammatory cells with the aid of extracellular vesicles (exo/ectosomes) [8,10,55,56] or by stromal synapses [22], rather than by direct contribution. Tcea1 is a major component of a chromatin transcription-enabling activity to potentiate transcription elongation through contiguous nucleosomes in a manner of acetyl-transferase p300 and acetyl-CoA dependence [57]. It implies that TCs have a low capacity of DNA damage and repair in the biological process, although the exact mechanisms remain unclear. Those changes of chromosome 1 genes can be considered as biomarkers, network biomarkers, or dynamic network biomarkers to monitor biological functions and involvements of TCs in physiological and pathophysiological condition [58,62].

In conclusion, the present study compared (for the first time) variations of chromosome 1 genes of pulmonary TCs from other neighbouring cells, *e.g*. MSCs, Fbs, AT II cells, ABCs, PACs, or bronchial and lung lymphocytes. Our data demonstrated about 25% or 70% genes of TC chromosome 1 were up- or down-regulated, respectively. Capn2, Fhl2 and Qsox1 were over-expressed mostly in TCs, by comparison with other cells. These findings indicate that biological functions of TCs are mainly associated with morphogenesis and local tissue homoeostasis. TCs may also participate in preventing the development of inflammation and fibrogenesis in chronic lung inflammation and as ‘local data suppliers’ for the immune response.

## Supporting information

**Figure S1** Details of the selected core network genes in telocytes isolated from the mouse lung and cultured for 5 days. **Figure S2** Details of the selected core network genes in telocytes isolated from the mouse lung and cultured for 10 days. **Figure S3** Details of the selected core network genes in mouse mesenchymal stem cells. **Figure S4** Details of the selected core network genes in mouse Fbs. **Figure S5** Details of the selected core network genes in mouse alveolar type II cells. **Figure S6** Details of the selected core network genes in mouse airway basal cells. **Figure S7** Details of the selected core network genes in mouse proximal airway cells. **Figure S8** Details of the selected core network genes in mouse lymphocytes from bronchial lymph nodes. **Figure S9** Details of the selected core network genes in mouse lung lymphocytes.

**Table S1** Gene expression omnibus database (GSE6846).

**Table S2** Details of chromosome 1 gene expression variations among different cell lines.

**Data S1** The profiles for entire genes.
