# Supplementary material for: Differences in the expression of chromosome 1 genes between lung telocytes and other cells: mesenchymal stem cells, fibroblasts, alveolar type II cells, airway epithelial cells and lymphocytes
Source: J Cell Mol Med. 2014 May 15;18(5):801–10. doi: 10.1111/jcmm.12302 (PMC4119386; doi:10.1111/jcmm.12302)
Supplement: Supplementary file 1 [file jcmm0018-0801-SD1.pdf]

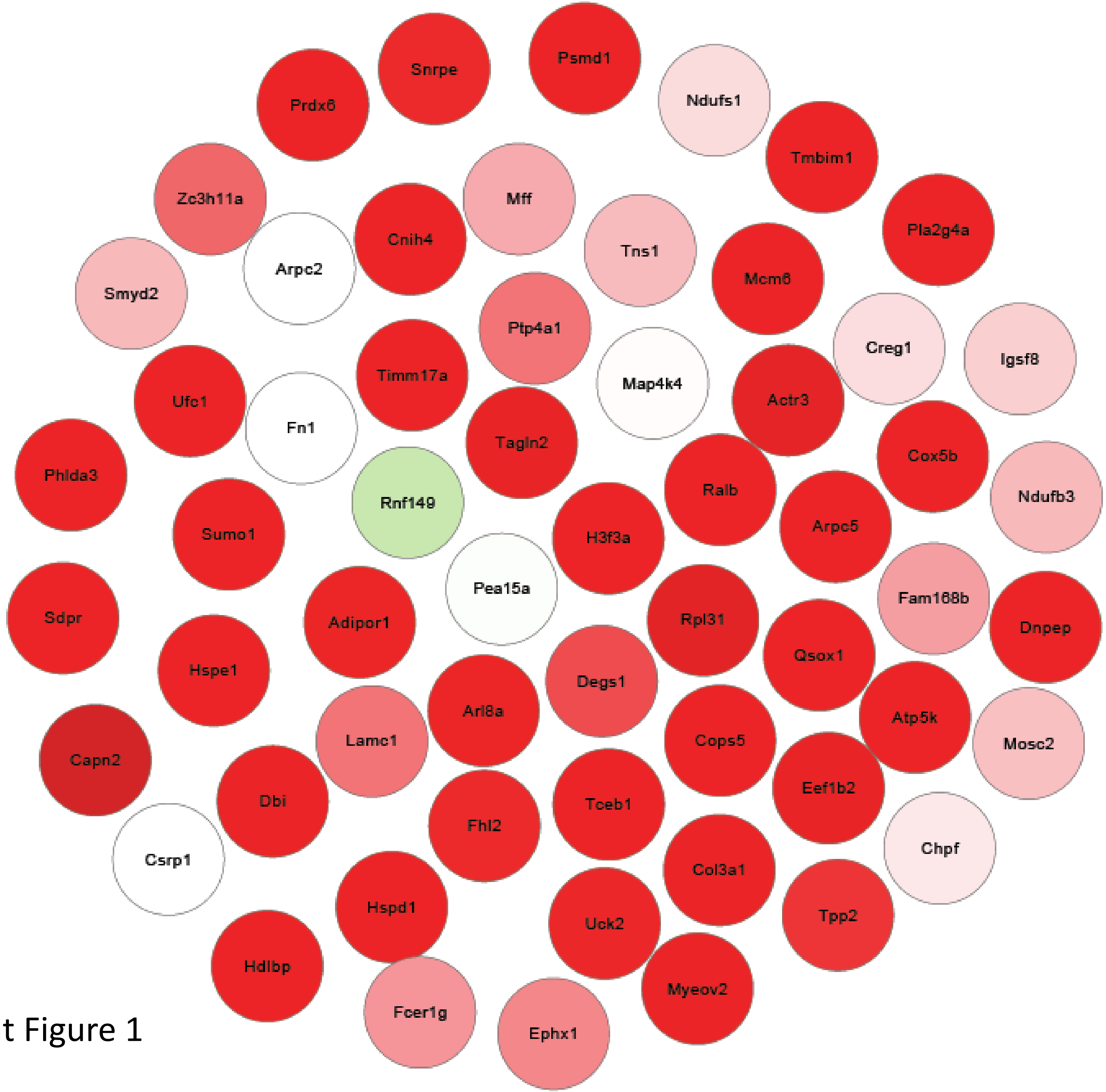

Supplement Figure 1

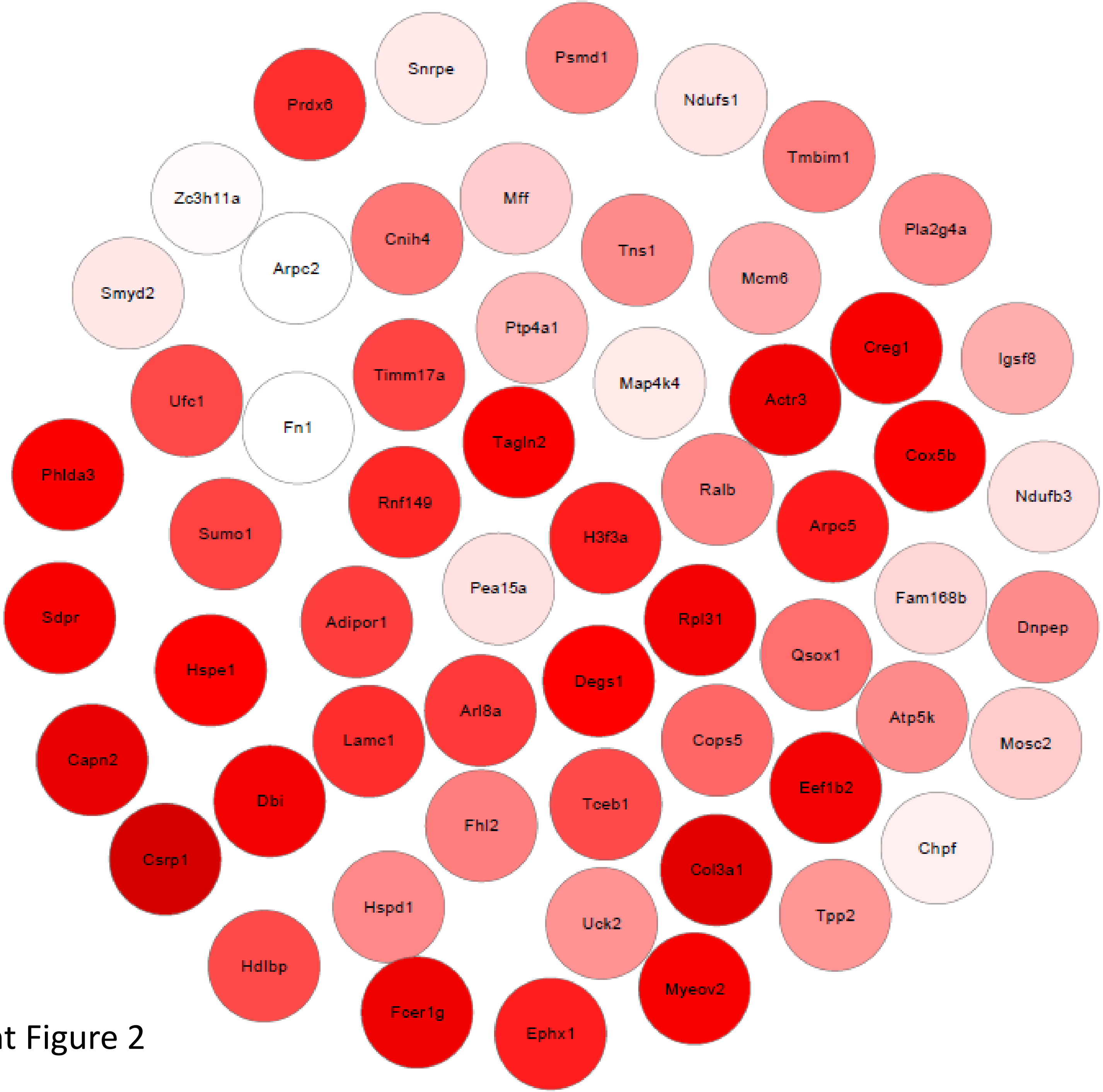

Supplement Figure 2

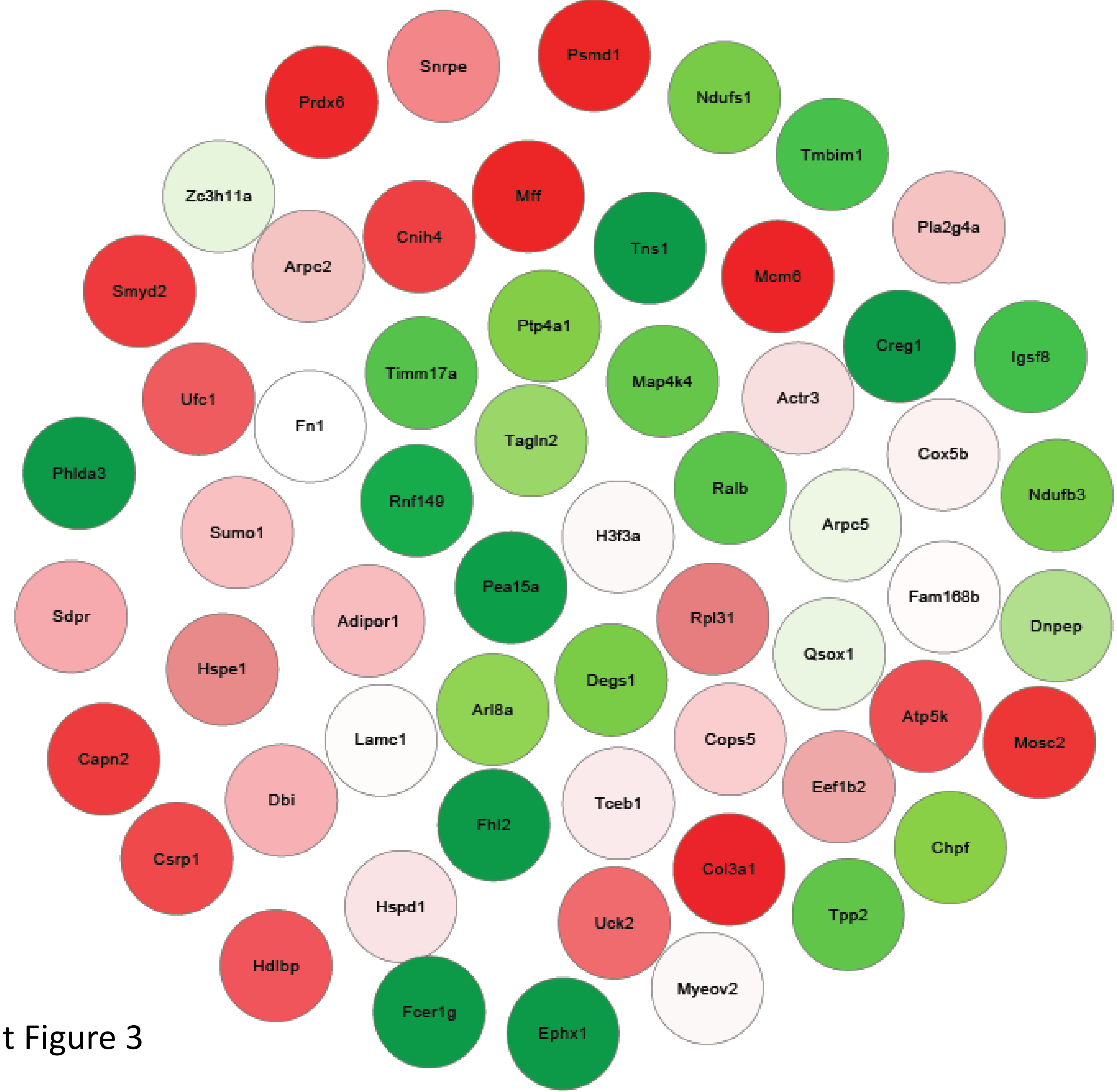

Supplement Figure 3

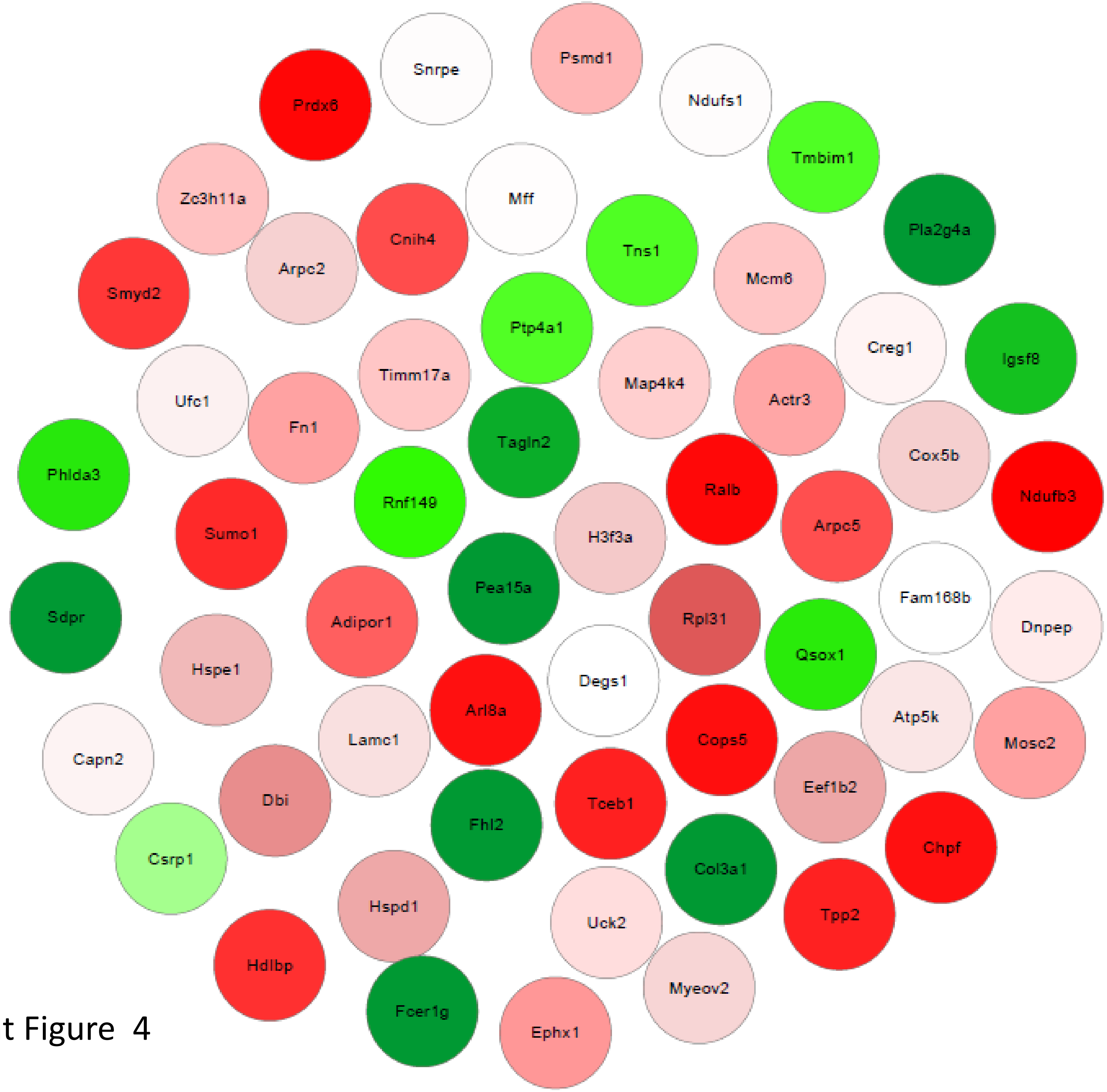

Supplement Figure 4

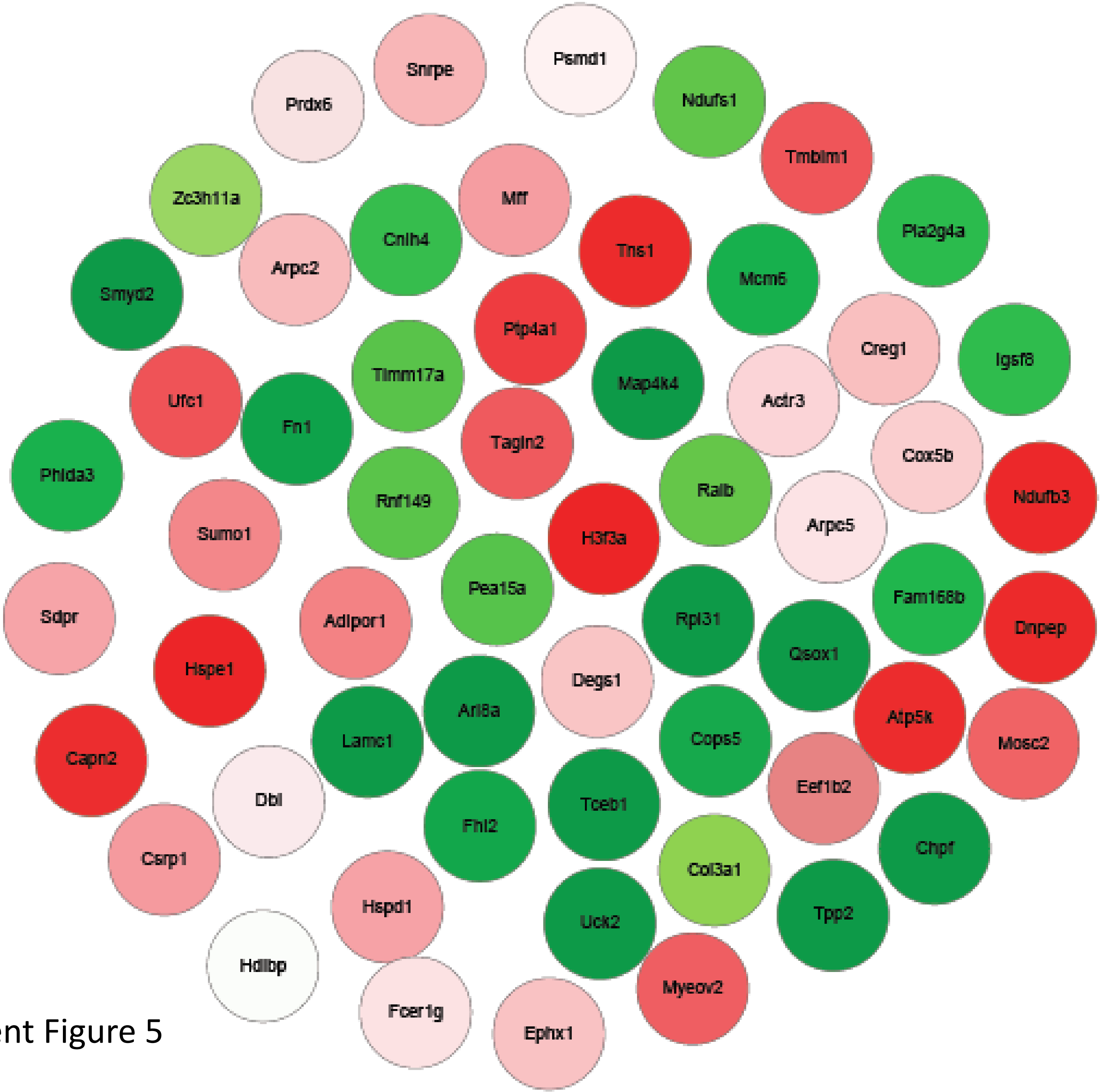

Supplement Figure 5

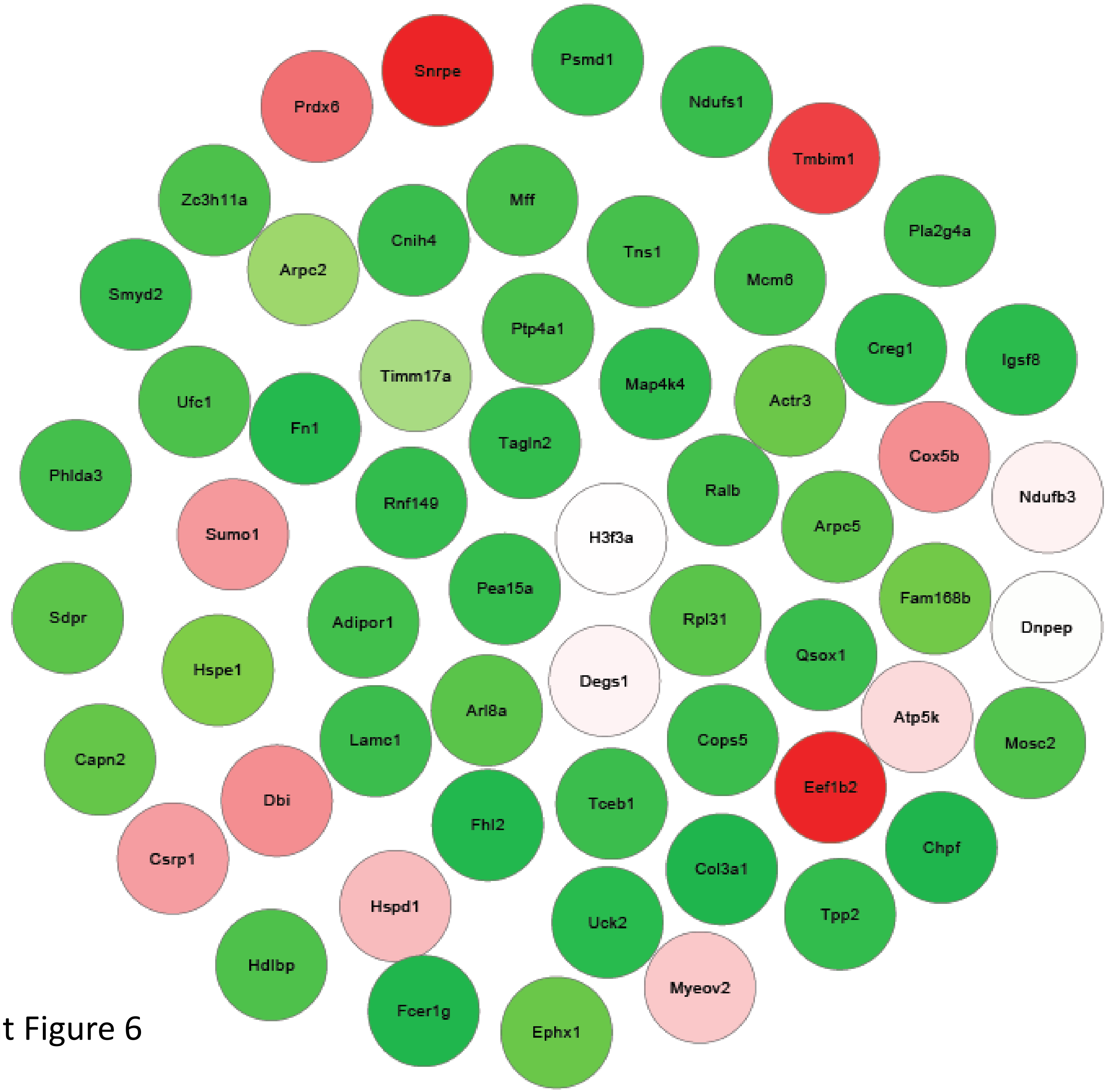

Supplement Figure 6

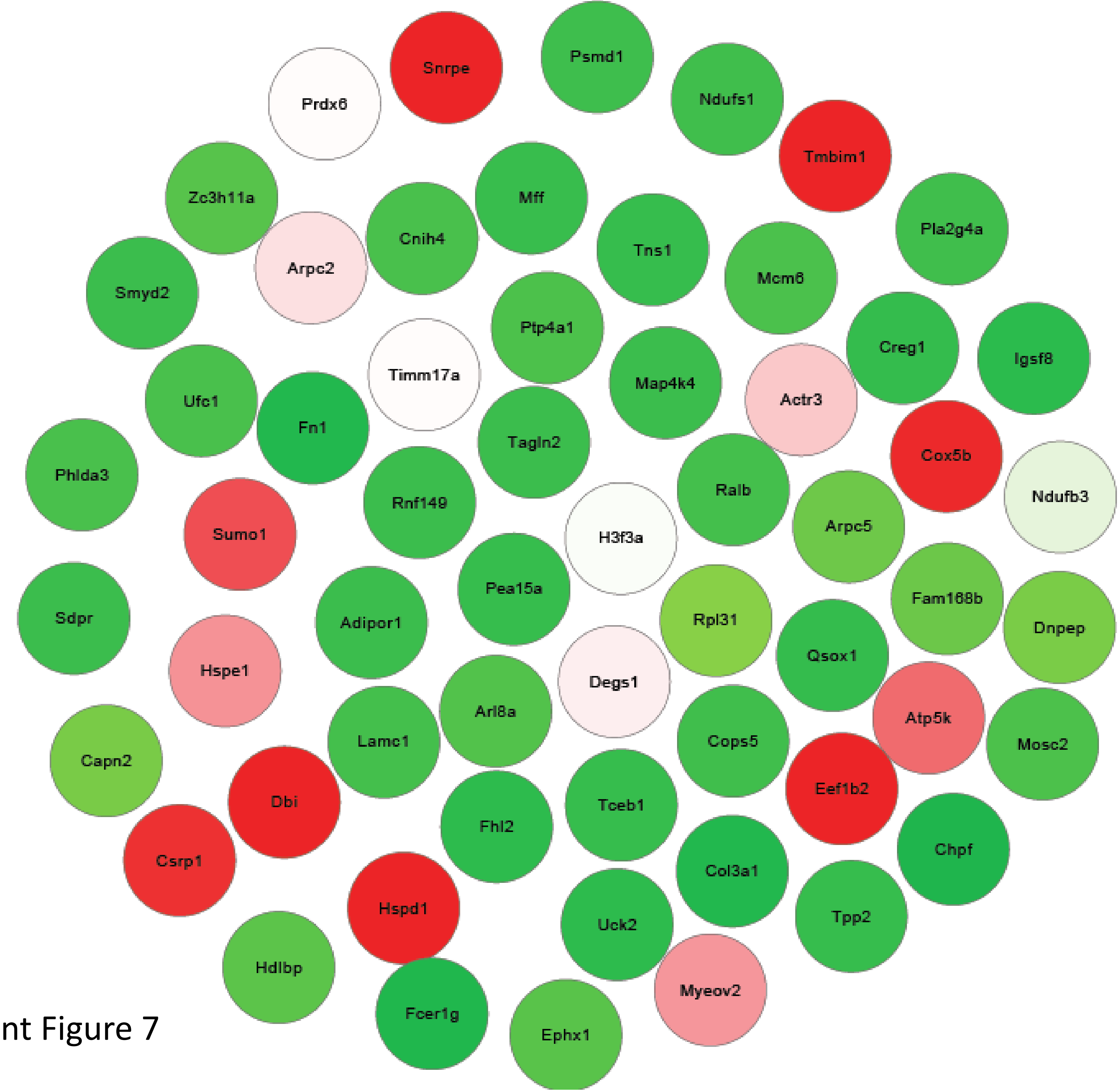

Supplement Figure 7

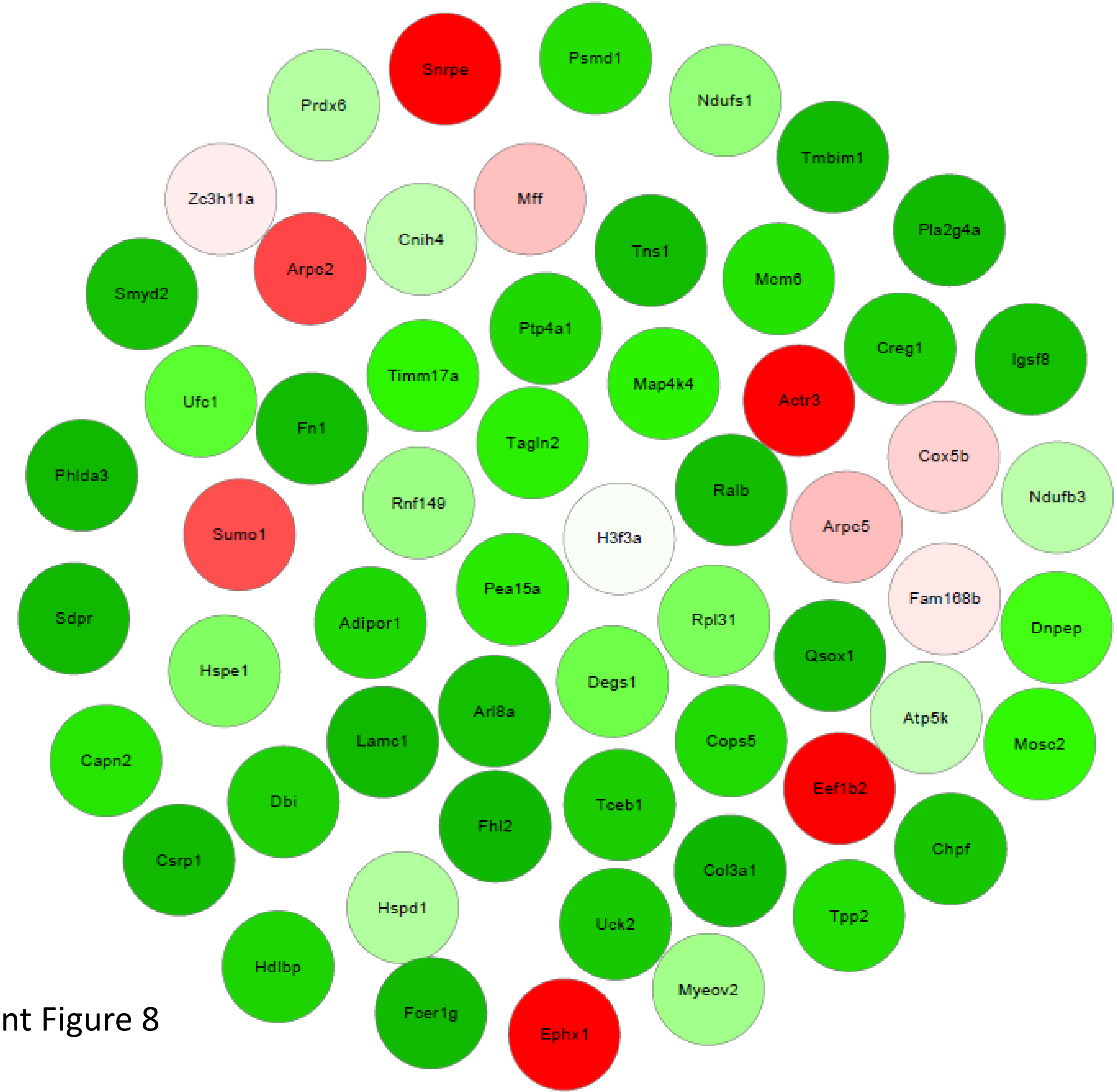

Supplement Figure 8

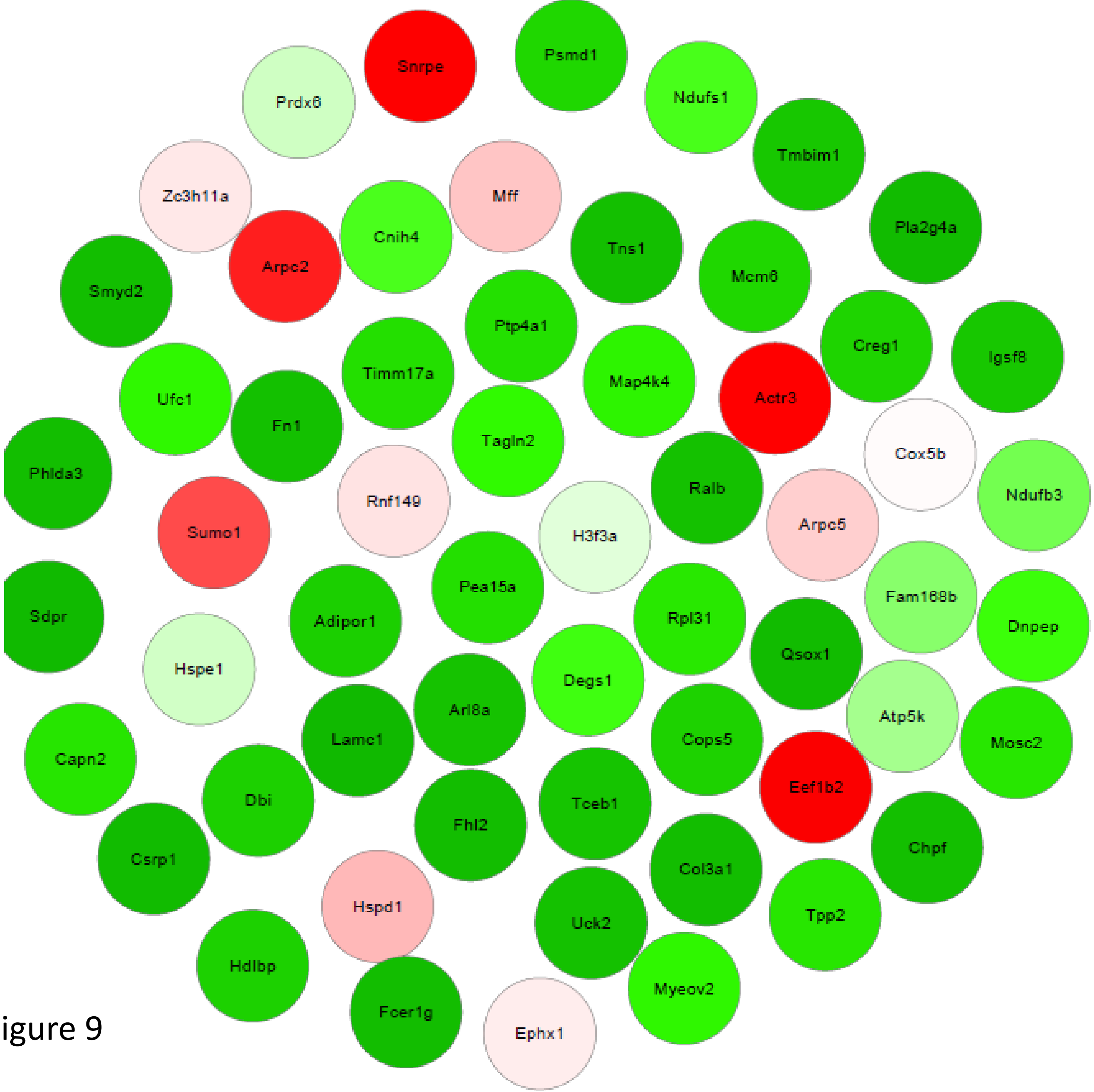

Supplement Figure 9
