# Supplementary material for: Differences in the expression of chromosome 1 genes between lung telocytes and other cells: mesenchymal stem cells, fibroblasts, alveolar type II cells, airway epithelial cells and lymphocytes
Source: J Cell Mol Med. 2014 May 15;18(5):801–10. doi: 10.1111/jcmm.12302 (PMC4119386; doi:10.1111/jcmm.12302)
Supplement: Supplementary file 5 — Data S1 The profiles for entire genes. [file jcmm0018-0801-SD5.docx]

Supplement Figure 1: Details of the selected core network genes in telocytes isolated from the mouse lung and cultured for 5 days.

Supplement Figure 2: Details of the selected core network genes in telocytes isolated from the mouse lung and cultured for 10 days.

Supplement Figure 3: Details of the selected core network genes in mouse mesenchymal stem cells.

Supplement Figure 4: Details of the selected core network genes in mouse Fbs.

Supplement Figure 5: Details of the selected core network genes in mouse alveolar type II cells.

Supplement Figure 6: Details of the selected core network genes in mouse airway basal cells.

Supplement Figure 7: Details of the selected core network genes in mouse proximal airway cells.

Supplement Figure 8: Details of the selected core network genes in mouse lymphocytes from bronchial lymph nodes.

Supplement Figure 9: Details of the selected core network genes in m
